# IFNAR(-/-) Mice Constitute a Suitable Animal Model for Epizootic Hemorrhagic Disease Virus Study and Vaccine Evaluation

**DOI:** 10.7150/ijbs.95275

**Published:** 2024-05-27

**Authors:** Luis Jiménez-Cabello, Sergio Utrilla-Trigo, Julio Benavides-Silván, Juan Anguita, Eva Calvo-Pinilla, Javier Ortego

**Affiliations:** 1 Centro de Investigación en Sanidad Animal (CISA), Instituto Nacional de Investigación y Tecnología Agraria y Alimentaria (INIA), Valdeolmos, 28130 Madrid, Spain.; 2 Instituto de Ganadería de Montaña (CSIC-Universidad de León), 24346 Grulleros, León, Spain.; 3 Centro de Investigación Cooperativa en Biociencias (CIC bioGUNE), 48160 Derio, Spain.; 4 Ikerbasque, Basque Foundation for Science, 48012 Bilbao, Spain.

**Keywords:** epizootic hemorrhagic disease virus, IFNAR(-/-) mouse, vaccine, bluetongue virus, viral coinfection

## Abstract

Epizootic hemorrhagic disease (EHD), caused by Epizootic hemorrhagic disease virus (EHDV), is an emerging and severe livestock disease. Recent incursion and distribution of EHDV in Europe have outlined the emerging character of EHD. Despite its worldwide impact, numerous knowledge gaps exist. A range of inconveniences restricts utilization of natural hosts of EHDV. Here, we show that adult mice deficient in type I IFN receptor (IFNAR(-/-)) are highly susceptible to EHDV-6 and EHDV-8 infection when the virus is administered subcutaneously. Disease was characterized by ruffled hair, reluctance to move, dehydration and conjunctivitis, with viraemia detected from day 5 post-infection. A deeper characterization of EHDV-8 infection showed viral replication in the lung, liver, spleen, kidney, testis and ovaries. Importantly, increased expression levels of pro-inflammatory cytokines IL-1β, IL-6 and CXCL2 were observed in spleen after EHDV-8 infection. Furthermore, IFNAR(-/-) adult mice immunized with a EHDV-8 inactivated vaccine elicited neutralizing antibodies specific of EHDV-8 and full protection against challenge with a lethal dose of this virus. This study also explores the possibilities of this animal model for study of BTV and EHDV coinfection. In summary, the IFNAR(-/-) mouse model faithfully recapitulates EHD and can be applied for vaccine testing, which can facilitate progress in addressing the animal health challenge posed by this virus.

## Introduction

Epizootic Hemorrhagic disease (EHD) is a *Culicoides*-transmitted disease of wild and domestic ruminants, mainly associated with cervid species such as white-tailed deer (*Odocoileus virginianus*) (WTD). It is included in both USA's National List of Reportable Animal Diseases and the notifiable disease list of the World Organization for Animal Health (WOAH) 1,2. Epizootic Hemorrhagic disease virus (EHDV), the causal agent of EHD, belongs to the genus *Orbivirus* within the family *Sedoreoviridae*. Similar to the highly related Bluetongue (BTV) and African horse sickness (AHSV) orbiviruses, up to seven different serotypes of EHDV have been described 3, with novel putative serotypes being recently identified 4,5. Since the first description and isolation of EHDV-1 in New Jersey in 1955 6, this virus has been isolated in all continents except Antarctica. This global distribution, along with a wide range of susceptible hosts, leads to a presumable significant annual worldwide economic impact 7 and jeopardizes wild ruminant populations. The upsurge of EHDV in regions where no cases were previously reported, like the recent incursion and spread in southern Europe (Spain, Italy, Portugal and France) of EHDV-8 8,9 or the northwards expansion in North America 10,11, as well as the increasing severity and prevalence among bovine populations, emphasize the relevance of this arboviral disease.

EHDV mainly affects wild ungulates, with WTD as the most affected host. This cervid species displays a high susceptibility to EHDV and significant mortality and morbidity rates are usually registered in North America 12,13. This marked susceptibility is maintained when experimental infections are conducted in WTD 14-22. Therefore, WTD has been the animal model preferentially used for the characterization of EHDV infection and disease, the study of virus pathogenesis, host defense mechanisms, transmissibility and vaccine research 8,14,17,19,23-26. Despite the invaluable possibilities that wildlife animal models offer, they pose several drawbacks mostly related with animal housing and handling, care and wellbeing 27. Traditional livestock experimental animals, e.g., cattle, sheep or goat, are also hosts of EHDV and an alternative to the WTD animal model. Nonetheless, there is marked inter- and intra-species diversity on pathogenicity and virulence among these domestic ruminant species 8. While goats are not susceptible to EHDV infection 28, sheep usually develop subclinical disease although viral replication occurs 28. Although EHDV outbreaks being usually characterized by mild or subclinical disease in bovine populations, recent EHDV outbreaks have been related with an increased pathogenicity among cattle populations in the affected areas 29. Cattle could solve some of the limitations of the WTD model. However, experimental infections of cattle with a wide range of EHDV isolates did not resemble the increased pathogenicity observed in the field in most cases 28,30-34, which hindered the implementation of this domestic animal for EHDV study. Nonetheless, it is worth noting that experimental infections with EHDV-6 and EHDV-7 isolates from Reunion Island and Israel, respectively, were successful in inducing clinical disease in cattle 34,35.

The study of viral disease pathogenesis and the development of effective therapies against viral diseases is supported by the utilization of suitable laboratory animal models. These offer a variety of advantages, including a reduction of costs and time, easy handling and affordable housing, and accessibility of a high number of optimal reagents. Immunocompromised mouse models are very useful for the study of a plethora of viral diseases 36. In this regard, mice deficient in the type I IFN (IFN-α/β) receptor (IFNAR(-/-)) have been stablished as a reliable animal model for two important orbiviruses, BTV and AHSV, as they reproduce the disease pathogenesis observed in natural hosts 37-40. The susceptibility to EHDV of the IFNAR(-/-) mouse model was assessed previously. A viral isolate of serotype 7 as well as inocula from spleen of infected cattle demonstrated some pathogenicity in this mouse model, although there is not consistent data on fundamental aspects of the susceptibility of this laboratory animal model to this viral disease 31,41.

In this work, we have studied the susceptibility of IFNAR(-/-) mice to different EHDV isolates. Moreover, we have determined the lethality of EHDV-6 and the European isolate of EHDV-8. We have then conducted a deep characterization of the infection with EHDV-8 in IFNAR(-/-) mice. Furthermore, we explored the possibilities of this mouse model to study orbivirus coinfections and test vaccine efficacy against EHDV.

## Results

### EHDV-6 and EHDV-8 cause lethal infection in adult IFNAR(-/-) mice

**Figure 1:**
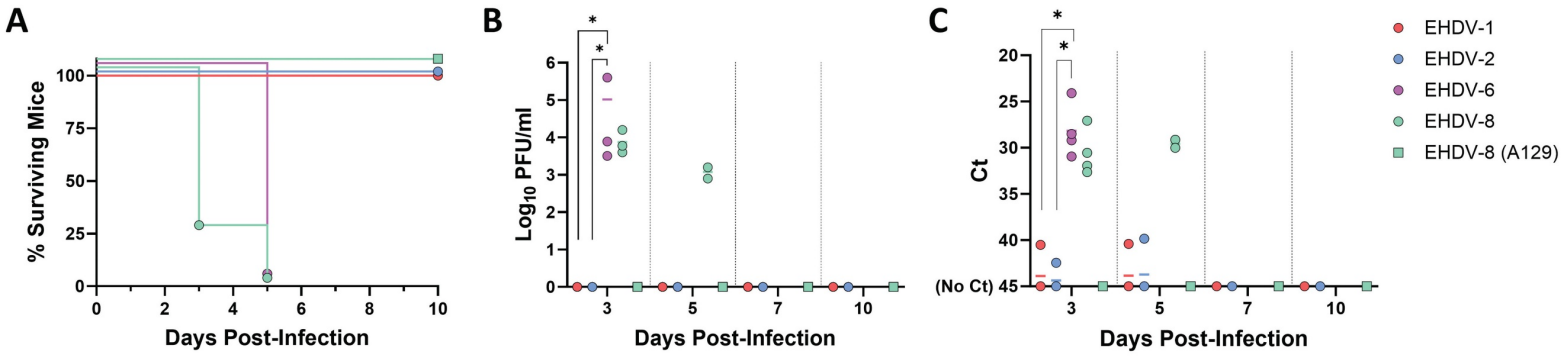
** Susceptibility of IFNAR(-/-) mice to EHDV infection.** Groups of IFNAR(-/-) mice (n=4) were subcutaneously inoculated with 10^4^ PFU of EHDV-1, EHDV-2, EHDV-6 or EHDV-8. A group of A129 mice was subcutaneously inoculated with 10^4^ PFU of EHDV-8. (A) Survival rates after infection. EHDV-6 and EHDV-8 survival curves were found statistically significant compared with EHDV-1, EHDV-2 and EHDV-8 (A129) survival curve as calculated by Log-rank test (P value < 0.05). (B) Titers calculated by plaque assay of EHDV-1, EHDV-2, EHDV-6 or EHDV-8 in blood of IFNAR(-/-) and A129 mice after viral inoculation. Points represent individual viral titer for each mouse and lines of the corresponding color represent the mean viral titer of each group. Differences between groups were calculated by multiple t test analysis using the Sidak-Bonferroni method. * P value < 0.05. (C) RNAemia analyzed by RT-qPCR after viral inoculation of IFNAR(-/-) and A129 mice. Expression of mRNA of segment 9 (encoding VP6 and NS4 proteins) was quantified at 3, 5, 7, 10, and 14 d.p.i. Results were expressed as Ct (left y axis). The real-time RT-qPCR specific for EHDV segment 9 was performed as described by Mann *et al.* 93. Points represent individual Ct for each mouse and lines of the corresponding color represent the mean Ct value of each group. “No Ct” values were considered as a Ct of 45 (as indicated in the Y-axis). Differences between groups were calculated by multiple t test analysis using the Sidak-Bonferroni method. * P value < 0.05.

The IFNAR(-/-) mouse model has been shown to be highly susceptible to BTV and AHSV infection 37-39. In contrast, little information on the susceptibility of this mouse model to EHDV exists 31,41. To stablish IFNAR(-/-) mice as a solid animal model for EHDV, we tested the susceptibility of IFNAR(-/-) mice to infection with different viral isolates of EHDV-1, EHDV-2, EHDV-6 and EHDV-8. Groups of male IFNAR(-/-) mice (n=4) were inoculated with a high dose (10^4^ PFU per mouse) of each virus. As stablished for BTV and AHSV infection of IFNAR(-/-) mice 40,42-45, the subcutaneous route of inoculation was chosen to partially mimic transmission through the bite of an infected Culicoides midge. After inoculation of EHDV-1 or EHDV-2, no clinical signs were observed in inoculated mice at any time post-inoculation. Further, no mortality was observed for these two inoculated groups (Fig. 1A). Consistently, the mice displayed undetectable levels of viraemia and RNAemia as measured by plaque assay in Vero cells or RT-qPCR, respectively (Fig. 1B,C). In contrast, inoculation with EHDV-6 or EHDV-8 caused marked clinical signs, e.g. ruffled hair, reluctance to move, dehydration and conjunctivitis, characteristic features of orbivirus infection in IFNAR(-/-) mice 38,40. Both serotypes of EHDV were highly lethal at this high dose of infection in this mouse model, with 100% mortality between days 3 and 5 post-infection (d.p.i.). (Fig. 1A). Viral replication was detected as we observed RNAemia and high titers of virus were isolated from blood at 3 and 5 d.p.i. for both inoculated groups (Fig. 1B,C). As a control, we also tested the susceptibility of A129 mice to infection with EHDV-8. After subcutaneous inoculation with 10^4^ PFU per mice of EHDV-8, these immunocompetent mice, with the same genetic background than IFNAR (-/-) mice, did not show any clinical sign or death following viral infection (Fig. 1A) nor viraemia or RNAemia after viral inoculation (Fig. 1B, C). These results demonstrate that, whereas inoculation with EHDV-1 and EHDV-2 do not lead to productive infection or disease, EHDV-6 and EHDV-8 are highly pathogenic in IFNAR(-/-) mice, causing clinical disease and death. Most importantly, these data show that type I interferon responses are critical for the control of EHDV infection.

### Determination of the lethal dose of EHDV-8 and EHDV-6 in IFNAR(-/-) mice

We then determined the lethal dose of EHDV-6 and EHDV-8 in IFNAR(-/-) mice. Groups of male IFNAR(-/-) mice (n=5) were subcutaneously inoculated with 10-fold dilutions of these two viruses (from 10 to 1000 PFU). Mice were monitored for survival, while viraemia and RNAemia were measured by plaque assay and RT-qPCR, as before.

**Figure 2:**
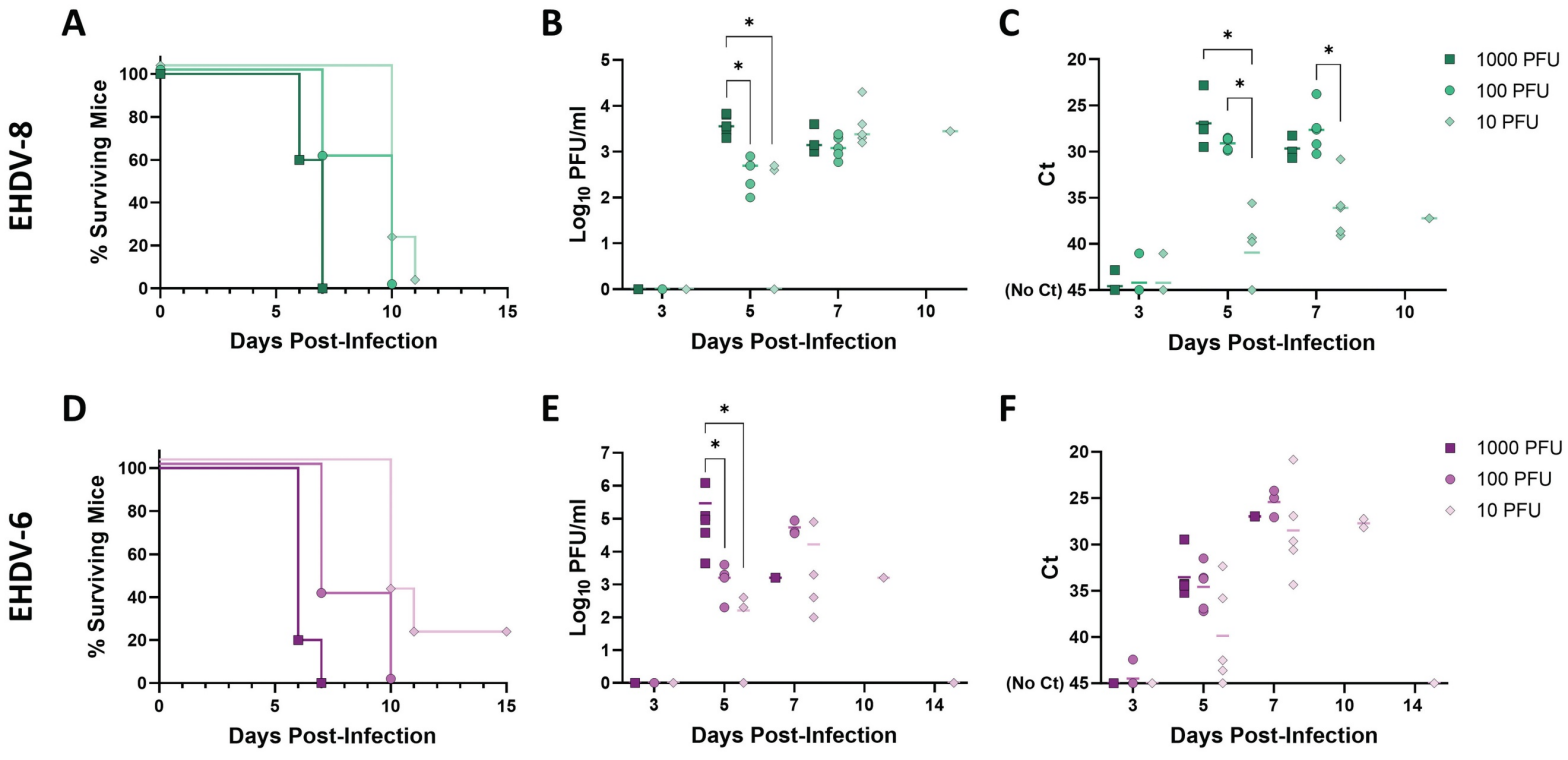
** Lethality determination of EHDV-8 and EHDV-6 in IFNAR(-/-) mice.** Groups of IFNAR(-/-) mice (n=5) were subcutaneously inoculated with 1000, 100 or 10 PFU of EHDV-8 (A, B, C) or EHDV-6 (D, E, F). (A,D) Survival rates after infection. Statistical differences were found between survival curves of EHDV-8 or EHDV-6 inoculation groups as calculated by Log-rank test (P value < 0.05). (B,E) Titers calculated by plaque assay of EHDV-6 or EHDV-8 in blood of IFNAR(-/-) mice after viral inoculation. Points represent individual log_10_ PFU/ml value for each mouse and lines of the corresponding color represent the mean log_10_ PFU/ml value of each group. Differences between groups were calculated by multiple t test analysis using the Sidak-Bonferroni method. * P value < 0.05. (C,F) RNAemia analyzed by RT-qPCR after viral inoculation of IFNAR(-/-) mice. Expression of mRNA of segment 9 (encoding VP6 and NS4 proteins) was quantified at 3, 5, 7, 10, and 14 d.p.i. Results were expressed as Ct (left y axis). The real-time RT-qPCR specific for EHDV segment 9 was performed as described by Mann *et al.* 93. Points represent individual Ct for each mouse and lines of the corresponding color represent the mean Ct value of each group. “No Ct” values were considered as a Ct of 45 (as indicated in the Y-axis). Differences between groups were calculated by multiple t test analysis using the Sidak-Bonferroni method. * P value < 0.05.

We observed a very similar lethality for both viruses. After inoculation with 1000 PFU of EHDV-6 or EHDV-8, clinical signs (ruffled hair, reluctance to move, dehydration and conjunctivitis) were observed as soon as 3 d.p.i., escalating in subsequent days until 6 and 7 d.p.i., when all mice belonging to these two inoculation groups succumbed to infection (Fig.2A, D). Some severely affected mice showed an enlargement of the testicular region. A delay in the onset of clinical signs (including the inflammation of the genital region) was observed for groups inoculated with 100 PFU of either EHDV-6 or EHDV-8, but all mice died between days 6 and 10 post-infection (Fig. 2A, D). Mice inoculated with 10 PFU did not show evidence of disease until day 7 post-infection. The severity of disease increased in subsequent days, leading to death of all mice inoculated with 10 PFU of EHDV-8 by 11 d.p.i. and 4 out of 5 mice belonging to the group inoculated with 10 PFU of EHDV-6 at 10 d.p.i. (Fig. 2A, D). Viraemia and RNAemia also showed a dose-dependent fashion (Fig. 2B, C, E, F). No virus or viral RNA could be detected at 3 d.p.i. but high levels of virus and RNA were observed in blood thereafter, peaking between days 5 and 7 post-infection. Importantly, one mouse inoculated with 10 PFU of EHDV-6 survived to infection (Fig. 2D). This mouse displayed detectable viraemia and RNAemia levels during the first 10 days-post-infection (Fig. 2E, F) but they were undetectable from 14 d.p.i. to 42 d.p.i. (end of the experiment, data not shown), which indicates that the viraemic and RNAemic status does not occur through a prolonged period. These results highlight the marked susceptibility of the IFNAR(-/-) mouse model to EHDV-6 or EHDV-8 infection. No differences were found in terms of virulence between these two EHDV serotypes in the IFNAR(-/-) mouse model.

### EHDV-8 replicates and produces histopathologic changes in target organs of infected IFNAR(-/-) mice

Next, we performed a deeper characterization of the infection with EHDV-8 in the IFNAR(-/-) mouse model. Two groups of male IFNAR(-/-) mice (n=5) were subcutaneously inoculated with a lethal dose (100 PFU) of EHDV-8. Animals were sacrificed at 4 or 6 d.p.i. A group of mice was used as control (mock-infected). Organs were harvested to determine the presence of viral RNA and to evaluate histopathological lesions.

**Figure 3:**
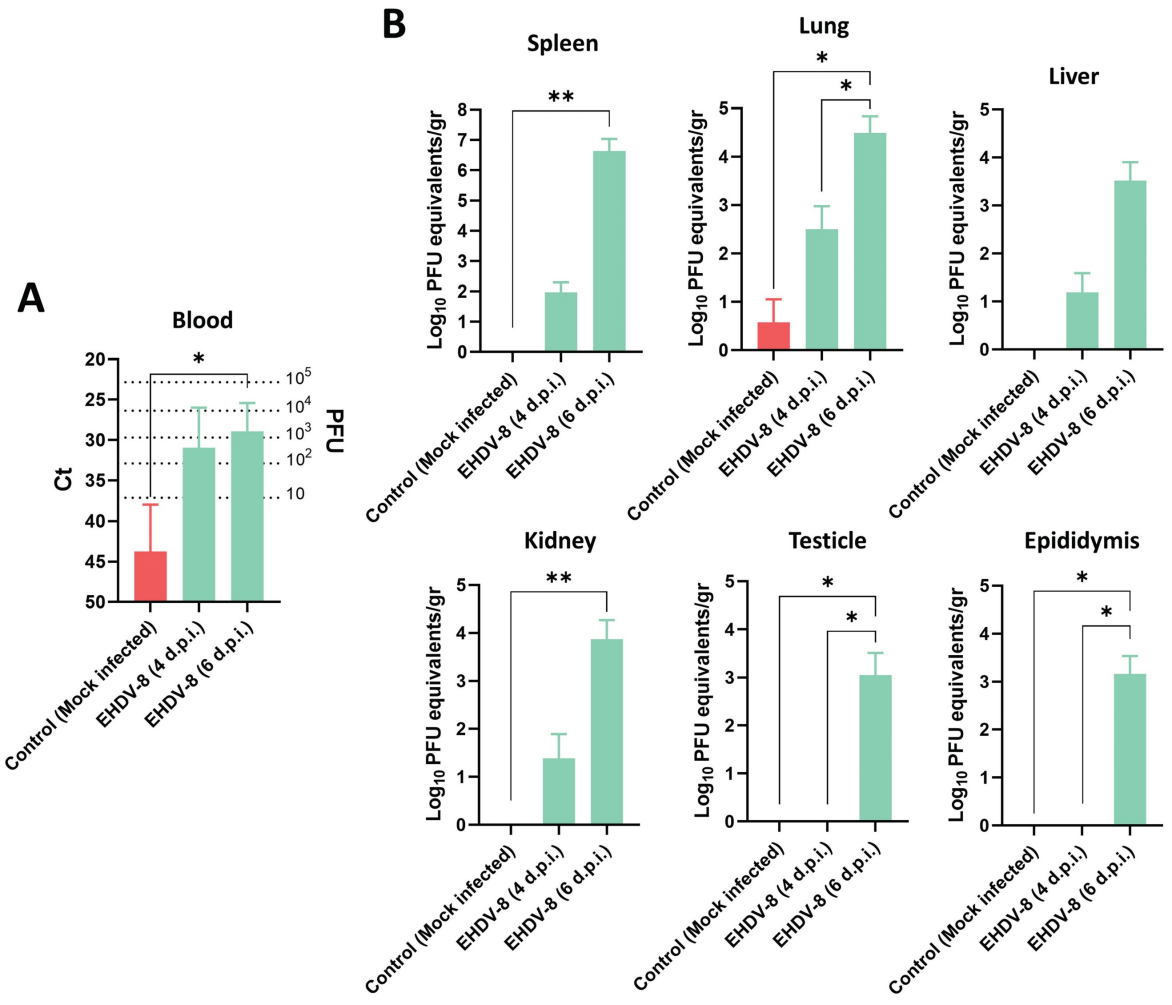
** Presence of viral RNA in EHDV-8 infected IFNAR(-/-) mice.** Two groups of IFNAR(-/-) mice (n=5) were subcutaneously inoculated with 100 PFU of EHDV-8. At 4 and 6 d.p.i., mice were euthanatized, and several organs were harvested for RNA extraction. A control group was included in the experiment. (A) RNAemia was analyzed and (B) the presence of virus was quantified in spleen, lung, liver, kidney, testicle and epididymis by RT-qPCR specific of mRNA of segment 9 (encoding VP6 and NS4 proteins). Different amounts of virus were titrated and used as standards to calculate PFU equivalents from Ct values. Results were expressed as (A) Ct (left y axis) and PFU equivalents (right y axis) or (B) Log_10_ PFU equivalents per gram of tissue (left y axis). The real-time RT-qPCR specific for EHDV segment 9 was performed as described by Mann *et al.* 93. Bars represent mean values of each group and error bars represent SD. Asterisks denote significant differences between groups (* p < 0.05; ** p < 0.0332, Kruskal-Wallis' test).

Viral RNA was detected in blood from day 4 post-infection (Fig. 3A), but clinical signs of disease were only observed in mice sacrificed at 6 d.p.i., coinciding with higher RNAemia levels (Fig. 3A). Spleen, lung, liver, thymus, heart, kidney, testicles and epididymis were harvested to determine the target organs of EHDV infection in IFNAR(-/-) mice. At day 4 post-infection, viral RNA was only detected by RT-qPCR in lung, liver, spleen and kidney of some inoculated mice (Fig. 3B). However, high levels of viral RNA were detected by RT-qPCR in these organs from all mice at 6 d.p.i. (Fig. 3B). No signal could be detected in the thymus and one single mouse displayed high levels of virus in the heart at 6 d.p.i., which could be due to the viral burden present in blood at this day (data not shown). Of note, some infected mice had enlarged testicular areas at day 6 post-infection and viral RNA could be detected in both testicular and epididymal tissues at this time point, while no signal was detected at 4 d.p.i. (Fig. 3B).

**Figure 4:**
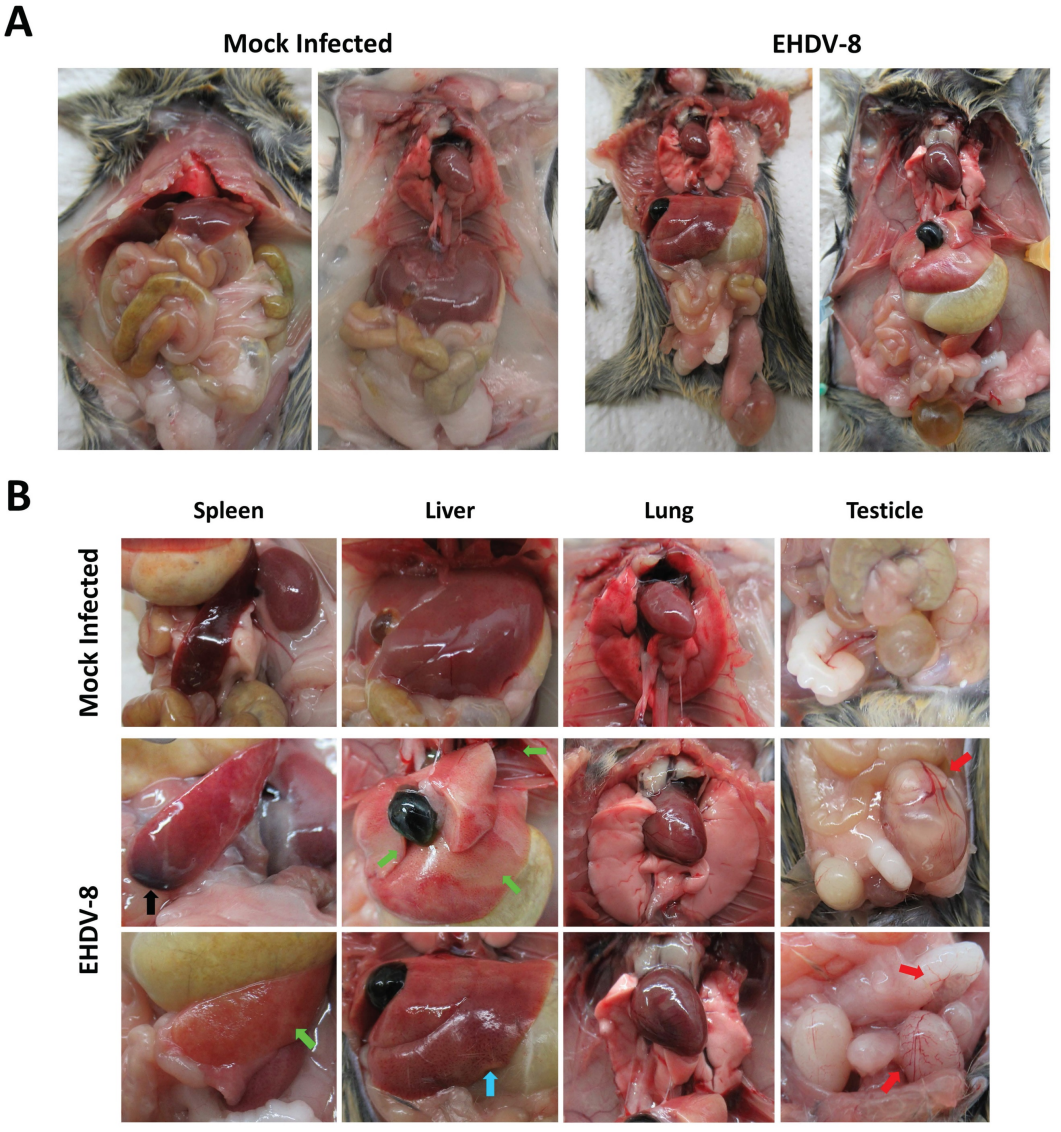
** Macroscopic analysis of EHDV-8 infected IFNAR(-/-) mice.** IFNAR(-/-) mice infected with EHDV-8 (100 PFU) were sacrificed at 6 d.p.i. and a necropsy was performed. (A) Whole-body images of non-inoculated and EHDV-8 inoculated mice. (B) Images of organs from non-inoculated and EHDV-8 inoculated mice. In spleen, splenomegaly was observed in infected mice as well as necrosis (black arrow) and discoloration (green arrow). Liver from infected mice evinced pale zones (green arrow) and increase of the organ size. Lung of infected mice did not display macroscopic lesions. Some mice infected with EHDV-8 showed enlarged testicles with an increased congestion (red arrows) in this organ but also in the seminal vesicle.

**Figure 5:**
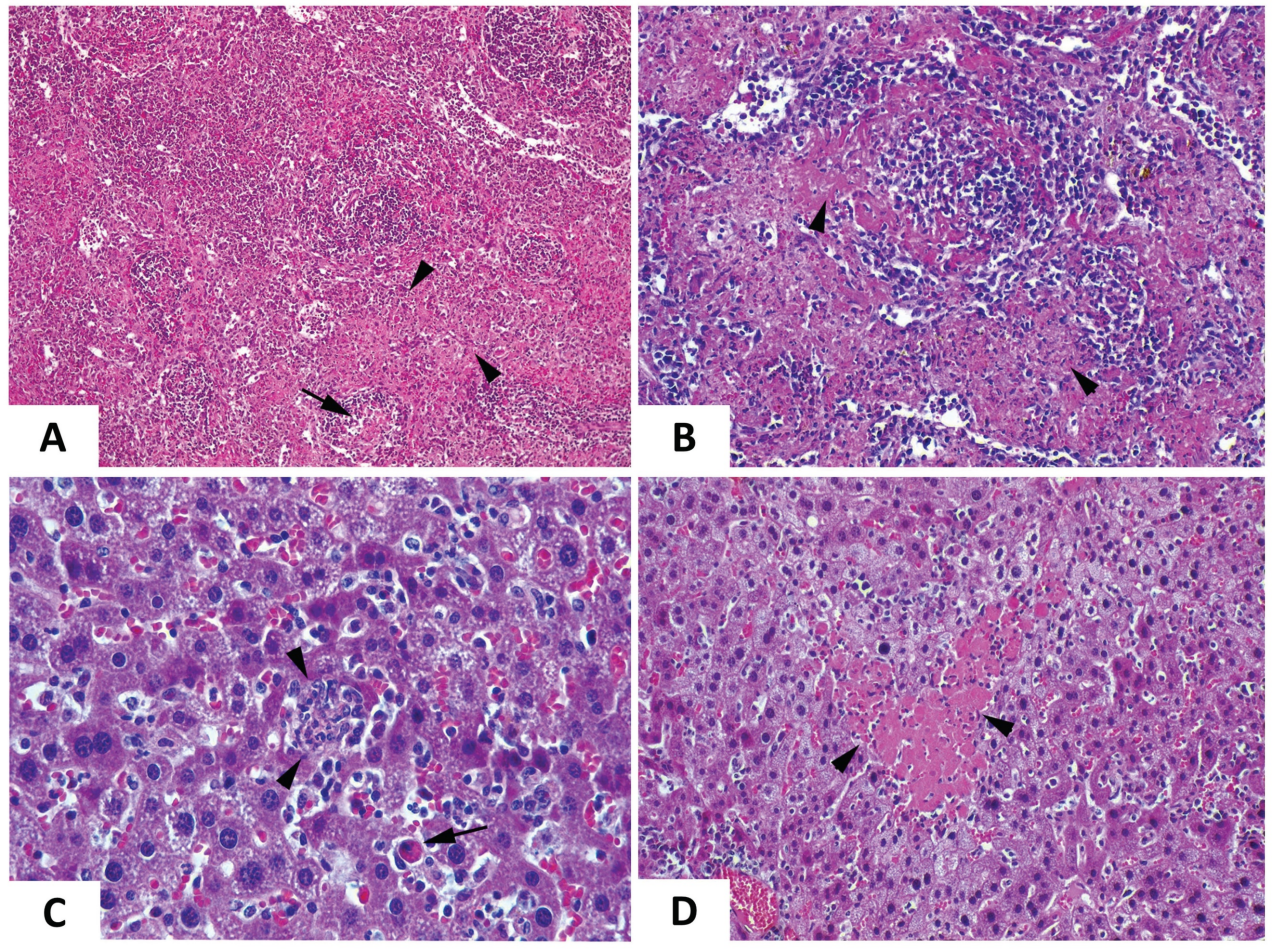
** Comparative photomicrograph of tissue sections from EHDV-8 infected and control IFNAR(-/-) mice.** Representative examples of the microscopic lesions found on the spleen (A,B) and the liver (C, D) of EHDV-8 infected IFNAR(-/-) mice at 6 d.p.i. (A). Severe necrotic splenitis denoted by diffuse and extensive fibrinoid necrosis of the red pulp (arrowheads) and depletion of lymphoid cells in the white pulp (arrows). H/E. Magnification 10x. (B) Perifollicular necrosis of the white pulp, accompanied by depletion of lymphoid cells (arrowheads). The loss of lymphocytes is substituted by accumulation of eosinophilic cellular debris, along with karyorrhectic remnants. H/E. Maginification 20x. (D) Non-purulent mild hepatitis characterized by infiltration of inflammatory cells, mainly lymphocytes, throughout the hepatic parenchyma, causing disorganization of the typical hepatocyte architecture. Small clusters of necrotic hepatocytes with shrunken cell bodies, accompanied by lymphocytic infiltration are scattered in the parenchyma (arrowheads). Additionally, individual necrotic hepatocytes with hypereosinophilic cytoplasm, and pyknotic nuclei are observed (arrow). There is also congestion of vessels and occasional phagocytosis of erythrocytes by macrophages is noted. H/E. Magnification 40x. (E) Sharply demarcated irregular patchy of coagulation necrosis (arrowheads) with mild infiltration of inflammatory cells. The parenchymal vessels show congestion. H/E. Magnification 20x.

The increase in RNAemia levels positively correlated with the presence of macroscopic changes in target organs, which were most evident at day 6 post-infection. Overall, inoculated mice displayed a broad edematous state (Fig. 4A) with macroscopic enlargement of the spleen, the liver and the gut (Fig. 4A,B). The spleen and the liver also exhibited a lighter color. Testicle and epididymis enlargement was also evident in one infected mouse. However, the histopathological analysis of testicles and epididymis revealed no signs of inflammation (data not shown). The mice also showed an active congestion of isolated vessels (Fig. 4A,B). Spleens were enlarged and brittle and showed areas with extensive tissue discoloration (Fig. 4B) while the liver displayed pale areas (Fig. 4B). The spleen also showed severe necrotic splenitis denoted by diffuse and extensive necrosis of the red pulp and depletion of lymphoid cells in the white pulp (Fig. 5A). We also observed perifollicular necrosis of the white pulp, accompanied by depletion of lymphoid cells. The loss of lymphocytes was substituted by accumulation of eosinophilic cellular debris, along with karyorrhectic remnants (Fig. 5B). Liver from infected animals showed non-purulent mild hepatitis characterized by infiltration of inflammatory cells, mainly lymphocytes, throughout the hepatic parenchyma, causing disorganization of the typical hepatocyte architecture. Small clusters of necrotic hepatocytes with shrunken cell bodies, accompanied by lymphocytic infiltration were scattered in the parenchyma. Additionally, individual necrotic hepatocytes with hypereosinophilic cytoplasm and pyknotic nuclei were observed. There was also congestion of vessels and occasional phagocytosis of erythrocytes by macrophages (Fig. 5C). We also noted sharply demarcated irregular patches of coagulative necrosis with mild infiltration of inflammatory cells. The parenchymal vessels showed congestion (Fig. 5D).

As EHDV has been isolated in vulvae of experimentally infected sheep 28, we studied the capacity of EHDV to replicate in the female reproductive tract. Female IFNAR(-/-) mice were inoculated with 100 PFU of EHDV-8 that, as males, showed severe clinical signs and similar RNAemia levels at 4 and 6 d.p.i. like those found in male IFNAR(-/-) mice. Furthermore, viral RNA was present at high levels in ovaries at 6 d.p.i. as measured by RT-qPCR (log_10_ mean PFU equivalents/gr value = 4.5444) (data not shown).

### EHDV-8 induces hematologic changes and a proinflammatory response

**Figure 6:**
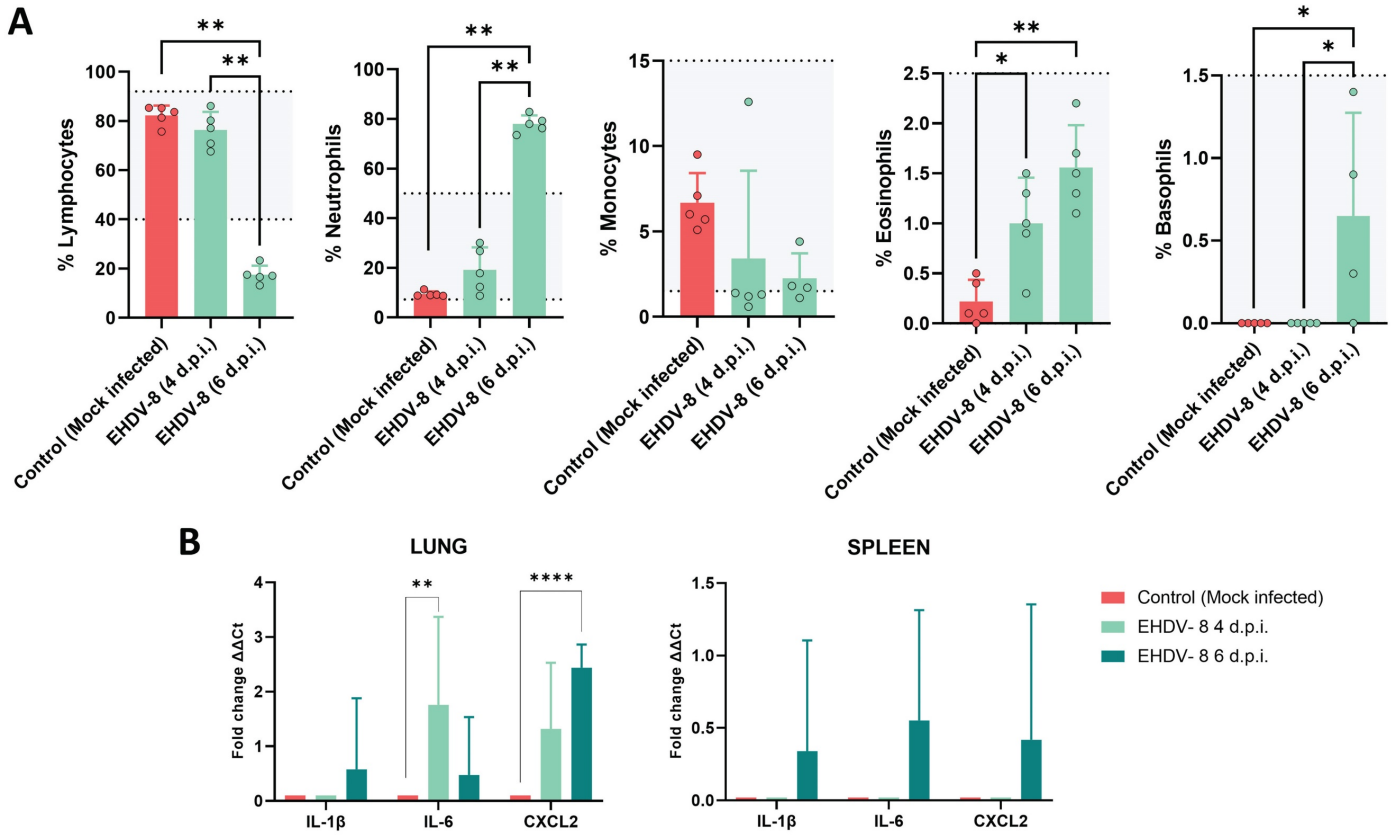
** Hematologic and proinflammatory cytokines changes in IFNAR(-/-) mice infected with EHDV-8.** (A) Blood was harvested from inoculated and mock-infected mice and analyzed in an autohematology analyzer (BC-5300 Vet; Mindray, China). The total percentage of lymphocytes, neutrophils, monocytes, eosinophils and basophils based on the total white blood cells were analyzed. Points indicate the individual value of each mouse, bars represent the mean value of each group and error bars represent SD. Dotted lines and grey area indicate the hematology normal adult mice reference range. Asterisks denote significant differences between groups (* p < 0.05; ** p < 0.0332, Mann-Whitney U test). (B) Total RNA from spleen, lung and liver were extracted, and the expression of mRNA of proinflammatory cytokines was quantified. The expression levels of the different genes were normalized to levels in mock-infected mice. Columns represent mean values and error bars represent the SD. Differences between groups were calculated by multiple t test analysis using the Sidak-Bonferroni method. ** p < 0.0332; **** p-value < 0.0001.

To further investigate the pathogenesis of EHDV in IFNAR(-/-) mice, changes in hematologic parameters were evaluated (Fig. 6A). A significant drop in lymphocyte percentages was observed after 6 d.p.i. compared to non-inoculated mice. Furthermore, the mice suffered neutrophilia at this same timepoint. Such differences were not observed at 4 d.p.i. compared to the control group. Whereas no differences were recorded regarding the percentage of monocytes between the inoculated and mock-infected groups, an increase in the total percentage of eosinophils and basophils were observed at 6 d.p.i., which could be related to the induction of an inflammatory response.

We also studied the expression of proinflammatory cytokines by measuring transcript levels in tissues where EHDV replicated. No upregulation of proinflammatory cytokine mRNAs were detected in liver, kidney, testicles or epididymis. In contrast, increased expression levels of the proinflammatory factors, interleukin-1β (IL-1β), interleukin-6 (IL-6) and CXC motif chemokine ligand 2 (CXCL2) were observed in the spleen at 6 d.p.i. (Fig. 6B). Similarly, transcription of IL-1β and CXCL2 was greatly increased at 6 d.p.i. in the lung, whereas the induction of IL-6 expression was higher at 4 d.p.i. than at 6 d.p.i. in this tissue (Fig. 6B). Overall, these results support that IFNAR(-/-) mice develop strong proinflammatory immune responses following EHDV-8 infection.

### IFNAR(-/-) mice as a model for BTV and EHDV coinfection

Since BTV and EHDV share a geographical distribution and susceptible animal hosts, and they are transmitted through common Culicoides species, simultaneous infections in wildlife and livestock are likely. We, therefore, assessed IFNAR(-/-) mice as a model for the study of coinfection with EHDV and BTV. We inoculated a group of male IFNAR(-/-) mice (n=5) with lethal doses of EHDV-8 (100 PFU) and BTV-1 (100 PFU). Control groups inoculated with either EHDV-8 or BTV-1 were included. After viral inoculation, mice were monitored daily for the appearance of disease, survival and RNAemia by RT-qPCR specific of BTV or EHDV.

**Figure 7:**
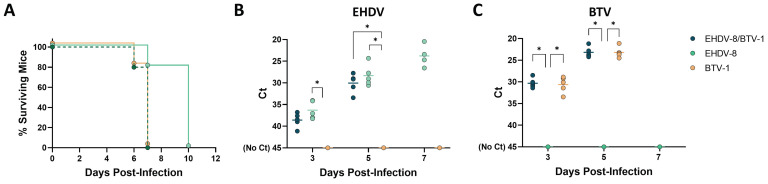
** Coinfection of IFNAR(-/-) mice with EHDV-8 and BTV-1**. A group of IFNAR(-/-) mice (n=5) was subcutaneously inoculated with 100 PFU of EHDV-8 and 100 PFU of BTV-1. Two additional groups were subcutaneously inoculated with 100 PFU of EHDV-8 or 100 PFU of BTV-1, respectively. (A) Survival rates after infection. Survival curves of EHDV-8/BTV-1 and BTV-1 inoculated mice were found statistically significant compared with survival curve of EHDV-8 inoculated mice as calculated by Log-rank test (P value < 0.05). (B) Detection of EHDV-8 RNA by RT-qPCR of IFNAR(-/-) mice after viral inoculation. Expression of mRNA of segment 9 (encoding VP6 and NS4 proteins) was quantified at 3, 5 and 7 d.p.i. Results were expressed as Ct (left y axis). The real-time RT-qPCR specific for EHDV segment 9 was performed as described by Mann *et al.* 93. (C) Detection of BTV-1 RNA by RT-qPCR of IFNAR(-/-) mice after viral inoculation. Expression of mRNA of segment 5 (encoding NS1 protein) was quantified at 3, 5 and 7 d.p.i. Results were expressed as Ct (left y axis). The real-time RT-qPCR specific for BTV segment 5 was performed as described by Toussaint *et al.* 94. Points represent individual Ct value for each mouse and lines of the corresponding color represent the mean Ct value of each group. “No Ct” values were considered as a Ct of 45 (as indicated in the Y-axis). Differences between groups were found by multiple t test analysis using the Sidak-Bonferroni method (* p < 0.05).

All coinfected mice died by day 7 post-infection (Fig. 7A). Individual inoculation with BTV-1 resulted in the same survival curve of the coinfection group. However, mice inoculated with EHDV-8 showed a delay in death, between 7 and 10 d.p.i. The survival curve of mice inoculated with EHDV-8 was significantly different to those of BTV-1 and EHDV-8/BTV-1 infection groups. Despite mortality might be impelled by the observed higher virulence of BTV-1 in IFNAR(-/-) mice, BTV and EHDV replication could be tracked in mice co-infected with these two viruses by RT-qPCR, as BTV and EHDV genomes were detected throughout the experiment. Importantly, no significant differences were found in terms of BTV or EHDV RNAemia levels between the coinfection group and both BTV and EHDV-control groups (Fig. 7B,C), which could indicate that there is not a synergy or interference in replication between this two orbiviruses. Overall, these data endorse the IFNAR(-/-) mouse model as a potential animal model for the evaluation of orbivirus coinfection.

### Immunized IFNAR(-/-) mice are protected against a lethal EHDV-8 challenge

**Figure 8:**
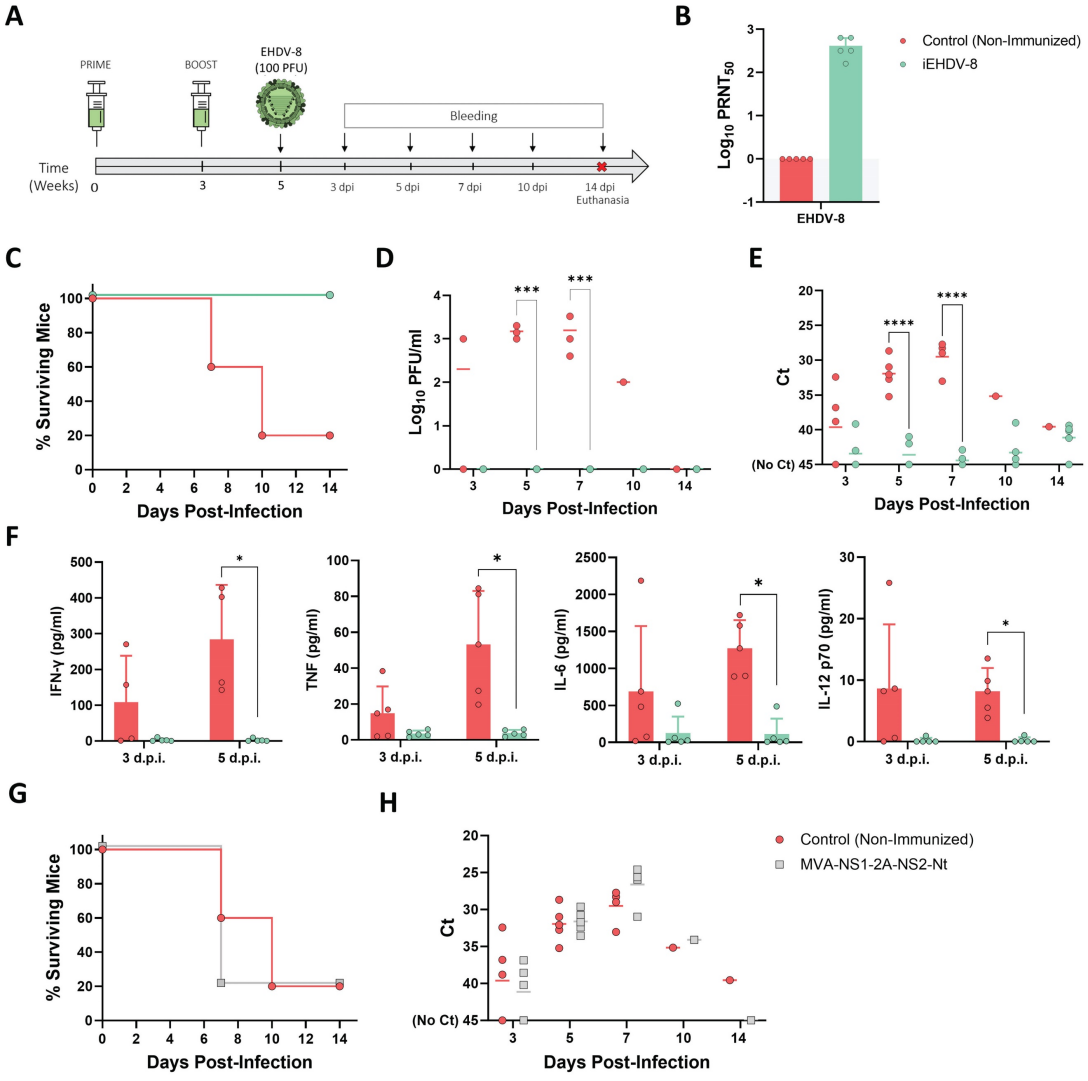
** Protection of immunized IFNAR(-/-) mice against a lethal challenge with EHDV-8.** (A) A group of IFNAR(-/-) mice (n=5) was intraperitoneally immunized with two doses (10^5^ PFU) of chemically inactivated EHDV-8. Another group was non-immunized (control). Two weeks post-boost, mice were challenged with 100 PFU of EHDV-8. (B) Neutralizing antibody titers against EHDV-8 in immunized animals by plaque reduction neutralization assay. Serum was extracted from blood samples harvested three weeks post-prime and three weeks post-boost. Cut-off: 0.69 (log_10_ 5). (C) Survival rates after infection. Survival curve of immunized mice was found statistically significant compared with survival curve of non-immunized mice as calculated by Log-rank test (P value < 0.05). (D) Viremia titers of IFNAR(-/-) mice after viral inoculation. Points represent individual log_10_ PFU/ml value for each mouse and lines of the corresponding color represent the mean log_10_ PFU/ml value of each group. Differences between groups were calculated by multiple t test analysis using the Sidak-Bonferroni method. *** p-value < 0.002. (E) RNAemia analyzed by RT-qPCR of IFNAR(-/-) mice after viral inoculation. Expression of mRNA of segment 9 (encoding VP6 and NS4 proteins) was quantified at 3, 5, 7, 10, and 14 d.p.i. Results were expressed as Ct (left y axis). The real-time RT-qPCR specific for EHDV segment 9 was performed as described by Mann *et al.* 93. Points represent individual Ct value for each mouse and lines of the corresponding color represent the mean Ct value of each group. “No Ct” values were considered as a Ct of 45 (as indicated in the Y-axis). Differences between groups were calculated by multiple t test analysis using the Sidak-Bonferroni method. **** p-value < 0.0001. (F) Cytokine production measured in sera from immunized and non-immunized mice at different time points post-infection. Points represent individual viral titer for each mouse, bars represent mean values of each group and error bars represent SD. Asterisks denote significant differences between immunized and non-immunized control mice (* p < 0.05) (The Mann-Whitney U test). (G,H) A group of IFNAR(-/-) mice (n=5) was intraperitoneally immunized with two doses (10^7^ PFU) of MVA-NS1-2A-NS2-Nt. Two weeks post-boost, mice were challenged with 100 PFU of EHDV-8. (G) Survival rates after infection. Survival curve of immunized mice was not found statistically significant compared with survival curve of non-immunized mice as calculated by Log-rank test (P value < 0.05). (H) RNAemia analyzed by RT-qPCR of IFNAR(-/-) mice after viral inoculation. Expression of mRNA of segment 9 (encoding VP6 and NS4 proteins) was quantified at 3, 5, 7, 10, and 14 d.p.i. Results were expressed as Ct (left y axis). The real-time RT-qPCR specific for EHDV segment 9 was performed as described by Mann *et al.* 93. Points represent individual Ct value for each mouse and lines of the corresponding color represent the mean Ct value of each group. “No Ct” values were considered as a Ct of 45 (as indicated in the Y-axis). No differences between groups were found (multiple t test analysis using the Sidak-Bonferroni method).

The feasibility of the IFNAR(-/-) mouse model to serve as a valid mean for preclinical vaccine research against BTV and AHSV has been confirmed 40,43,45. We thus evaluated the potential use of this mouse model for vaccine efficacy evaluation against EHDV. A group of adult male IFNAR(-/-) mice (n=5) was immunized with two doses (10^5^ PFU) of Alum adjuvanted chemically inactivated EHDV-8 by intraperitoneal injection in a three-week interval (Fig. 8A). A group was left untreated (control). Two weeks after the booster dose, mice were subcutaneously challenged with a lethal dose (100 PFU) of EHDV-8. Subsequently, mice were monitored for survival and viremia and RNAemia were analyzed by plaque assay and RT-qPCR, respectively.

Immunized animals did not display any evidence of disease nor RNAemia detected by RT-qPCR (data not shown) at any timepoint after the prime or boost immunization with the chemically inactivated EHDV-8 (inactivated virus was previously passaged three times in Vero cells to ensure complete virus inactivation). High titers of neutralizing antibodies (nAbs) against the homologous EHDV-8 were observed right before viral challenge (two weeks post-boost) (Fig. 8B). After challenge, non-immunized control mice (with the exception of one mouse) succumbed to infection between 7 and 10 d.p.i. (Fig. 8C) with viraemia and RNAemia levels rising from 3 d.p.i. and peaking during 5 and 7 d.p.i. (Fig. 8D,E). In contrast to the control group, immunization with two doses of the inactivated EHDV-8 elicited a 100% survival rate (Fig. 8C) with absence of clinical signs of disease and undetectable viraemia and RNAemia throughout the experiment (Fig. 8D,E). We measured circulating levels of proinflammatory cytokines in control and immunized IFNAR(-/-) mice sera. Non-immunized animals displayed an increase in IFN-γ, TNF, IL-6 and IL-12 after inoculation with EHDV-8, with an important and significant increase at day 5 post-infection compared to the immunized group (Fig. 7F). Undetectable circulating levels of these proinflammatory cytokines were registered in sera from immunized mice at any time post-infection (Fig. 7F), indicating that immunized mice were full protected and no viral replication happens after infection with EHDV-8.

Recently, we engineered a recombinant vaccine candidate based on modified vaccinia Ankara (MVA) viral vector co-expressing BTV nonstructural proteins NS1 and NS2-Nt 45. This vaccine candidate confers full protection against challenge with several BTV serotypes when applied in a prime-boost regime. To determine whether this experimental BTV vaccine could cross-protect against EHDV, we immunized a group of IFNAR(-/-) mice (n=5) with two doses of 10^7^ PFU of MVA-NS1-2A-NS2-Nt at a three-week interval. Two weeks after the booster dose, mice were subcutaneously challenged with a lethal dose (100 PFU) of EHDV-8. Immunized mice succumbed to infection at 7 d.p.i., with one mouse surviving, similarly to what was observed in the non-immunized control group (Fig. 7G). Immunized animals displayed a RNAemia profile comparable to that observed in the control group throughout the experiment (Fig. 7H), indicating that this recombinant BTV vaccine candidate based on the nonstructural proteins NS1 and NS2-Nt of BTV does not induce cross-protection against EHDV-8.

## Discussion

Reliability of mouse models articulates the basis to study crucial aspects of infectious diseases and underpins the efficacy of preventive and therapeutic approaches. Research of veterinary viral diseases is restricted by a variety of factors mostly related to the availability and maintainability of natural host species. Although natural hosts are ideal for studies of pathogenesis and vaccine testing, and findings in murine models should be carefully extrapolated to natural host species, the implementation of the IFNAR(-/-) mouse model for BTV and AHSV has facilitated research on host-virus interactions, virulence, immunobiology, and preclinical studies of vaccine efficacy. Given the emergence and increased pathogenicity among livestock of EHDV, we have characterized the IFNAR(-/-) mouse model for the *in vivo* study of this arboviral disease.

EHDV can infect newborn mice 46,47. However, this option is unsustainable and vaccine evaluation would require mature animals to mount adaptive immune responses. Considering the experience with BTV and AHSV 37,38,40, the IFNAR(-/-) mouse model was coherently assessed for EHDV susceptibility, showing a marked sensitivity to a viral isolate of EHDV-7 31,41. Here, we evaluated four different isolates of EHDV corresponding to serotypes 1, 2, 6 and 8. Viral isolates EHDV-1 USA1955/01 6 and EHDV-2 CAN1962/01 30 did not productively infect IFNAR(-/-) mice. Conversely, these immunocompromised animals were highly susceptible to viral isolates of EHDV-6 (EHDV-6/MOR2006/07) and EHDV-8 (EHDV-8/Spa). The reasons of these differences on virulence and pathogenicity in the IFNAR(-/-) mouse model are uncertain. In wildlife, viral isolates EHDV-1 USA1955/01 and EHDV-2 CAN1962/01 are markedly virulent in WTD 20,21 but cattle and other farm animals do not show such susceptibility to them 28,30,34. In contrast, the EHDV isolates of serotype 6 and 8 used here were causal agents of recent outbreaks in the Mediterranean basin and these events were characterized by a significant pathogenicity in cattle populations 8. Moreover, Eschbaumer and colleagues also demonstrated that a recent isolate of serotype 7 (EHDV-7/ISR2006/13), also highly pathogenic in cattle, was able to productively infect IFNAR(-/-) mice causing significant disease and death 31,41. Innate immune responses are crucial against viral pathogens and IFNs are a critical component of this first line of antiviral defense 48. It has been shown that host genetics differences related to type I IFN and inflammatory cytokine responses can influence EHDV disease outcome 23. As BTV 49,50, EHDV is a potent type I IFN inducer 17, whose up-regulation can inhibit viral spread and determines disease progression in the infected host 17,51. Notwithstanding other virulence factors, the differential virulence observed in cattle between the viral isolates used in this work could be partly explained by a differential immunomodulation of the host IFN response, which eventually might or not restrict host range. Although it can help explain the inability of EHDV-8 to infect A129 mice, the differential immunomodulation of the host IFN response cannot explain our results in IFNAR(-/-) mice, whose susceptibility to other orbiviruses such as BTV depends on an impaired IFN-I response 50,52. Therefore, other virulence determinants must be involved in the differential virulence between these viruses. For BTV, extensive passage in cell culture is related with an attenuation of the virulence 53,54. Considering that the record of cell culture passages of EHDV-1 USA1955/01 and EHDV-2 CAN1962/01 is undetermined, this differential virulence may be due to a likely attenuation of these viruses caused by extensive passage in cell culture through the years. Nonetheless, it is important to note that, since their isolation in the mid-20th century 6,21, both EHDV isolates have been shown to be nonpathogenic in a wide variety of ruminant species even when used as highly virulent inoculums from WTD 8,55. The virulence of recent EHDV-1 and EHDV-2 isolates should be evaluated in the IFNAR(-/-) mouse model. A recent isolate of EHDV-1 from a 2016 outbreak in Israel produced mild or asymptomatic disease in cattle 56. Regarding EHDV-2, recent outbreaks in USA in 2012 were associated with clinical illness in cattle although authors concluded that environmental variables had greater influence on the severity of this outbreak rather than viral genetic changes 57. In any case, the differences observed in this work seem to correlate with the differential pathogenicity in cattle observed between primal and late EHDV isolates, which eventually reflects the utility of this mouse model for the study of pathogenicity and virulence of EHDV as for BTV 54.

For serotype 7 of EHDV (EHDV-7/ISR2006/13), disease outcome and progression are dose-dependent in IFNAR(-/-) mice, although viral replication occurs in absence of clinical signs when a low infectious dose is used 41. As stated by the authors 41, the course of disease after EHDV-7 infection of IFNAR(-/-) mice is similar to AHSV, which induces transient viraemia and clinical signs when a low infectious dose is inoculated but whose severity dramatically increases at higher doses 40. On the contrary, the IFNAR(-/-) mouse model usually shows a marked susceptibility to BTV infection. Even at low doses, inoculation of this animal model with BTV-1, BTV-4 (MOR2009/09, BTV-4M) and BTV-8 leads to high mortality although viral replication and disease progression varies according the inoculated dose 37,38,50. Here, we saw that EHDV-6 and EHDV-8 infection of IFNAR(-/-) mice is lethal even at low infectious doses, but virological parameters as well as disease progression are dose-dependent. Moreover, subclinical disease does not occur unlike after EHDV-7 inoculation. Therefore, the course of disease after inoculation of EHDV-6 or EHDV-8 is more similar to BTV than to AHSV. Nonetheless, this varying virulence between EHDV-7 and EHDV-6 or EHDV-8 is also a characteristic of BTV infection of IFNAR(-/-) mice. Whereas BTV-1, BTV-4M and BTV-8 are lethal in the IFNAR(-/-) mouse model, inoculation with BTV-4 (Spain/01) or BTV-9 (Italian strain) is more comparable to AHSV 38,53. Hence, differential virulence between serotypes and strains is a feature of orbivirus infection.

For infectious disease research, one of the most important aspects of a reliable laboratory animal model is its capacity to reproduce or, at least, mimic the disease of study. For this reason, we conducted a deep characterization of the infection of IFNAR(-/-) mice with the emerging EHDV-8. As for BTV, after primary replication in lymphoid tissues, EHDV circulates through the blood stream. In the case of the most studied EHDV host, WTD, and cattle, animals usually become viraemic starting at day 3 or 4 post-infection. Thereafter, viraemia levels peak between days 5 and 7 post-infection 16-19,22,31,33,35,58 followed by viral clearance in surviving animals during subsequent weeks. Nevertheless, the existence of prolonged viraemic status is a very common feature in natural hosts of EHDV 17,19,22,31,35. Recently, Spedicato M, *et al.* (2023) conducted experimental infection of cattle and sheep with a European isolate of EHDV-8, showing that RNAemia also peaks at 7 d.p.i. coinciding with the onset of clinical signs. Animals remained positive for viral RNA for a long period 59. Here, we observed an identical viraemia and RNAemia profile in IFNAR(-/-) mice inoculated with EHDV-6 and EHDV-8 although no long-term viraemia or RNAemia seems to be an aspect of EHDV infection of IFNAR(-/-) mice. After primary replication and spread through the blood stream, the virus colonizes different organs. In experimental infections of WTD and other cervid species, the virus has been isolated in lymphoid tissues and other organs such as cerebrum, cerebellum, heart, lung and skin 14,15,19,32,58,60. EHDV has also been detected in spleen, lung, liver and kidney in cattle and in spleen of sheep 33,35,59,61. In IFNAR(-/-) mice, we saw that spleen (and probably other lymphatic tissues such as lymph nodes, similar to what is observed for BTV 38) is one of the main target organs for EHDV replication. Eschbaumer *et al.* also detected infectious virus in the spleens of IFNAR(-/-) mice after inoculation with EHDV-7. Moreover, they observed necrotic foci in the liver, likely caused by viral replication 41. Aside from spleen, we also identified other EHDV-target organs shared between IFNAR(-/-) mice and natural EHDV hosts such as the liver, lung and kidney, where the virus replicates. Macroscopic lesions in the liver or spleen were also characteristic of EHDV-8 infection in IFNAR(-/-) mice. Moreover, virus replication was associated with histopathological lesions in these tissues. Overall, viral and pathological features of EHDV in IFNAR(-/-) mirror those found in severely affected natural hosts of this viral disease.

Important aspects of the pathogenesis induced by EHDV in WTD are reflected in the IFNAR(-/-) mouse model. One of the hallmarks that characterizes EHDV and other orbiviral infections is the presence of lymphopenia and neutrophilia in infected animals 15-19,22,32,62. These two hematologic features were present in EHDV-infected IFNAR(-/-) mice, positively correlating with viral replication and disease severity. The lesions of the spleen may also explain this lymphoid depletion. Another important aspect that significantly contributes to the pathogenesis caused by EHDV infection in natural hosts is the induction of the so-called cytokine storm 63. EHDV replication in macrophages and endothelial cells triggers the release of proinflammatory cytokines such as IL-1β and IL-6, exacerbating inflammation that leads to tissue injury adding to the direct viral damage caused by replication in the endothelium of infected WTD 14. The uncontrolled expression of these two cytokines is positively associated with disease severity in other viral infections, being a hallmark of cytokine storms 64-67. EHDV-infected IFNAR(-/-) mice displayed elevated circulating levels of IL-6 as well as augmented transcription levels of IL-1β and IL-6 in the spleen and lung, tissues where EHDV replicates at high levels. Inflammation and subsequent tissue damage could be also exacerbated in infected IFNAR(-/-) mice by increased expression of the neutrophil chemoattractant CXCL2 68. Importantly, the augmented expression levels of CXCL2 positively correlated with neutrophilia. Often, neutrophil recruitment contributes to the induction and worsening of the immunopathology associated to cytokine storms during viral infections 69, which could be associated also with EHDV infection. Excessive production of other proinflammatory cytokines such as IFN-γ, TNF, and IL-12 may also be involved in exacerbated inflammation leading to tissue damage and mortality of EHDV-8 infected mice, similarly to other viral diseases 70,71. Mice also showed a general state of marked congestion, which could be indicative of inflammation in the vascular endothelium 66. In summary, the IFNAR(-/-) mouse model resembles important aspects of disease pathogenesis which may facilitate the understanding of the pathogenesis and virulence of EHDV.

As stated previously, EHDV has been detected in tissue samples from a diversity of organs in natural hosts, including the male reproductive system 15. A conspicuous feature of EHDV infection in IFNAR(-/-) mice was the marked enlargement of the testicular area. Although not all male IFNAR(-/-) mice inoculated with EHDV displayed this remarkable inflammation, viral RNA was uniformly detected in testicles and epididymis among infected animals. These two male reproductive organs constitute “immune privilege” sites, which are characterized by the existence of both blood-testis-barrier (BTB) and blood-epididymis-barrier (BEB), along with a particular immunological environment 72,73. Thus, our results suggest that systemic EHDV infection can lead to penetration of these immunological barriers and, consequently, to viral replication in both organs. Although this kind of tissues has not been extensively analyzed in natural EHDV hosts, severe hemorrhagic lesions were observed in the epididymis and the testicular parenchyma of WTD severely infected with EHDV 15. The absence of lesions in these tissues of some IFNAR(-/-) mice can be explained by a belated viral replication in these organs. Animals infected with 100 PFU of EHDV-8 tend to succumb to infection between days 7 and 10 post-infection. We detected viral RNA in testis and epididymis not earlier than day 6 post-infection, very near to death, which may avert the progress of tissue damage in some cases. However, the usage of this mouse model can shed light on the impact of EHDV in livestock fertility.

Transmissibility of EHDV can be also explored by utilization of the IFNAR(-/-) mouse model. As EHDV, the prototypical BTV is mainly an arbovirus transmitted by Culicoides midges, but it can be transmitted by alternative routes 74. For instance, transmission through semen containing infectious virus has been experimentally confirmed, and the re-emergence of BTV-8 in France in 2015 may be originated by this route 75,76. The presence of viral RNA in the male reproductive tract of infected IFNAR(-/-) mice indicates the feasibility of this model to explore this transmission route for EHDV. Further, vertical transmission is also observed for BTV 74. For EHDV, viral RNA was detected in the vulvae of a viraemic sheep 28. Besides, the virus has been described as abortifacient in cattle 77. Here, we observed that EHDV-8 replicated at high levels in the female genital tract. Considering that viral infection of the ovaries compromises pregnancy 78,79, the IFNAR(-/-) mouse model could shed light on the underlying mechanisms of this phenomenon. This model also provided new insights into oral transmission of BTV 80, even though evidence of horizontal transmission of BTV existed 81. Oral and fecal shedding of EHDV by natural hosts also occurs, which could be further studied in IFNAR(-/-) mice. In essence, this immunocompromised mouse model can provide new perspectives into vertical, venereal, direct, oral and mechanical transmission of EHDV, and its consequences regarding male and female fertility.

IFNAR(-/-) mice are a useful and effective tool for efficacy studies of novel vaccines against BTV and AHSV 36,82-84. Here, we confirmed the usefulness of the IFNAR(-/-) mouse model for the definition of effective vaccine candidates against EHDV. As no commercial vaccine is currently available for EHDV-8, we used alum adjuvanted chemically inactivated EHDV-8 for immunizations. Importantly, all immunized animals developed strong homologous nAbs titers, which indicates that IFNAR(-/-) mice can mount adaptive responses against EHDV, and that binary ethyleneimine (BEI) inactivation do not alter neutralizing epitopes of EHDV-8 viral particles. The protection mediated by the inactivated whole EHDV-8 vaccine in IFNAR(-/-) mice infected with a lethal dose of EHDV-8 was complete. Hypercytokinemia was completely abrogated in immunized mice, with absent IL-6 expression. In this sense, lower IL-6 expression is linked to disease resistance of some subspecies of WTD 14. Thus, the absence of immunopathology may be also an indicative of virus neutralization induced by the vaccine. Shared B- and T-cell epitopes and cross-reactive responses between BTV and EHDV are known 85,86. We evaluated the protective potential against EHDV of a recombinant MVA-based vaccine against BTV. Our results indicate that no cross-protection against EHDV is afforded by the MVA-expression of BTV NS1 and NS2-Nt proteins. In any case, these data together with the complete protection induced by the inactivated EHDV-8 vaccine reinforce the relevance of the IFNAR(-/-) mouse to accurately ascertain vaccine efficacy. Considering the experience with BTV and AHSV, it is expected that protective efficacy of EHDV vaccine formulations in IFNAR(-/-) mice will correlate with that in natural hosts, although further work is still needed to confirm this.

Viral coinfection among non-related arboviruses has occurred between EHDV and West Nile virus 87. However, the most likely coinfection event involving EHDV implies the prototypical BTV, as they share significant epidemiological similarities. In this work, we made a first approach to study EHDV and BTV coinfection *in vivo*. Our focus was to determine whether we could assess virological parameters for both virus in IFNAR(-/-) mice and, if so, any interaction existed between these two viruses in this mouse model. Viral coinfection can potentially leads to different scenarios 88. The most common virus-virus interaction is interference, which implicates the suppression of viral replication of one virus 88,89. A synergistic effect involves a promotion of viral replication 90, although it is not as common as viral interference. It seems that viral noninterference characterizes coinfection between EHDV-8 and BTV-1 in IFNAR(-/-) mice. However, this phenomenon is mostly related to viral coinfection between viruses that do not share tissue tropism 88, which is not the case of EHDV and BTV. The absence of a perceivable effect on virulence or viral replication between BTV-1 and EHDV-8 may rely on the marked virulence of both viruses, especially BTV-1. Distinguishable effects may arise between different serotypes and strains of BTV and EHDV. An extensive analysis involving a several of serotypes and strains of both viruses should be considered. Furthermore, the utilization of non-lethal doses or non-virulent viruses, or even application of different times of inoculations, may offer a more valuable approach to study the impact of potential interactions between EHDV and BTV *in vivo*. Anyway, we demonstrated that both viral infections are easily tracked with previously designed versions of RT-qPCR, avoiding the need for building a new system. Importantly, virus-virus interactions in coinfection cases can depend on the immunomodulation of the type-I IFN response 91, which could explain the noninterference between EHDV-8 and BTV-1 in IFNAR(-/-) mice. Although this can constrain the extrapolation of data on coinfection in IFNAR(-/-) mice to natural hosts of EHDV, the implementation of IFNAR(-/-) mice for EHDV and BTV coinfection studies can potentially forward research related to virus-virus interaction, host-virus interaction, virus transmission and evolutionary dynamics, as well as offer novel possibilities for dual BTV/EHDV vaccine evaluation.

In summary, we have characterized a small animal model for EHDV infection based on adult IFNAR(-/-) mice. This animal model reproduces many aspects observed in EHDV natural hosts during infection. The availability of a reliable laboratory animal model for EHDV, such as the IFNAR(-/-) mouse model, will expand research possibilities beyond those achievable with natural hosts and will undoubtedly enhance the advance in the development of novel EHDV vaccines as for BTV and AHSV 40,43,45.

## Materials and Methods

### Cells lines and viruses

Green monkey kidney cells (Vero) (ATCC, Cat. No. CCL-81) and BHK-21 cells (ATCC; catalog no. CCL-10) were grown in Dulbecco's Modified Eagle's medium (DMEM) (Biowest, Nuaillé, France) supplemented with 2mM glutamine (Gibco, Waltham, MA, USA) and 5% FCS (Gibco, Waltham, MA, USA).

BTV serotype 1 (ALG2006/01) (BTV-1), EHDV serotype 1 (EHDV-1) (USA 1955/01), EHDV serotype 2 (EHDV-2) (CAN 1962/01), EHDV serotype 6 (EHDV-6) (EHDV-6 MOR2006/07) and EHDV serotype 8 (EHDV-8/Spa) (isolated in Spain, 2022) were used in the experiments. EHDV-8 Spanish isolate was isolated from cattle blood in KC insect cells and passaged twice in BHK cells. Viruses were passaged once in KC insect cells and virus-working stocks were grown in BHK cells. Virus stocks and titrations were performed by standard methods previously described 92.

### Mice

Male and female type I interferon receptor defective mice (IFNAR (-/-)) on A129 Sv/Ev background and A129 mice were used throughout. All mice were matched for age (8 weeks, and 6 months, respectively). Mice were housed under pathogen-free conditions and allowed to acclimatize to the biosafety level 3 (BSL3) animal facilities at the Animal Health Research Center (CISA-INIA, CSIC), Madrid, before use.

### Mice experiments

First, to assess the susceptibility of IFNAR(-/-) mice to the different EHDV isolates used in this study, groups of male IFNAR(-/-) mice (n=4) were subcutaneously inoculated with 10^4^ PFU of EHDV-1, EHDV-2, EHDV-6 or EHDV-8. A group of A129 mice (n=4) was subcutaneously inoculated with 10^4^ PFU of EHDV-8. Mice were examined for survival and clinical signs daily. Submandibular blood collection was carried out at 3, 5, 7, and 10 d.p.i. for the analysis of viremia by plaque assay and RT-qPCR.

Second, to determine the lethal dose of EHDV-8 and EHDV-6, groups of male IFNAR(-/-) mice (n=5) were subcutaneously inoculated with 10, 100 or 1000 PFU of EHDV-8 or EHDV-6. Mice were examined for survival and clinical signs daily. Submandibular blood collection was carried out at 3, 5, 7, 10 and 14 d.p.i. for the analysis of viremia by plaque assay and RT-qPCR. Blood samples from a surviving mouse inoculated with 10 PFU of EHDV-6 were collected for 6 weeks.

Third, for the *in vivo* characterization of EHDV-8 infection, two groups of male IFNAR(-/-) mice (n=5) were subcutaneously inoculated with a lethal dose of EHDV-8 (100 PFU). A group of mice (n=5) was included as a control (non-infected). One group of inoculated mice was sacrificed at 4 d.p.i and the other inoculated group was sacrificed at 6 d.p.i. Two female IFNAR(-/-) mice were infected with 100 PFU of EHDV-8 and sacrificed at 6 d.p.i. Submandibular blood collection was carried out at the day of sacrifice for the analysis of viremia by RT-qPCR. Spleen, lung, liver, thymus, heart, kidney, testicles, epididymis and ovaries were harvested from mice. Tissues were homogenized in phosphate-buffered saline (PBS) using a Tissue Lyser homogenizer (Qiagen). The detection of viral RNA was measured by RT-qPCR.

Finally, to assess viral coinfection with EHDV and BTV, a group of male IFNAR(-/-) mice (n=5) was subcutaneously inoculated with lethal doses of EHDV-8 (100 PFU) and BTV-1 (100 PFU). Two groups of male IFNAR(-/-) mice mice (n=5) were included as control of infection with EHDV-8 or BTV-1, respectively. Mice were examined for survival and clinical signs daily. Submandibular blood collection was carried out at 3, 5 and 7 d.p.i. for the analysis of viremia by RT-qPCR.

### Mice immunization and challenge

A group of male IFNAR(-/-) mice (n=5) was intraperitoneally immunized following a homologous prime-boost regime consisting of two doses of 1x10^5^ PFU per mouse of chemically inactivated EHDV-8 in Imject® Alum (Thermo Fisher Scientific), administered three weeks apart. EHDV-8 was inactivated by incubation with 3 mM binary ethyleneimine (BEI) (Merck). Thereafter, inactivated virus was passaged three times in Vero cells to confirm the total inactivation prior to mice immunization. A group of mice (n=5) was left untreated (control). Animals were subcutaneously challenged with a lethal dose of EHDV-8 (100 PFU) two weeks post-boost. Submandibular blood collection was carried out in mice every week after the prime and boost doses. Sera of immunized and control animals were collected three weeks post-prime and two weeks post-boost for the analysis of the neutralizing response. After virus challenge, mice were daily examined for survival and clinical signs, and blood samples were collected at 3, 5, 7, 10 and 14 d.p.i. for the analysis of viremia and RNAemia by plaque assay in Vero cells and RT-qPCR, respectively. Sera of immunized and control animals were collected at 0, 3, 5 and 7 d.p.i. for the analysis of circulating proinflammatory cytokines.

An additional group of male IFNAR(-/-) mice (n=5) was intraperitoneally immunized following a homologous prime-boost regime consisting of two doses of 1x10^7^ PFU per mouse of MVA-NS1-2A-NS2-Nt administered three weeks apart. Animals were subcutaneously challenged with a lethal dose of EHDV-8 (100 PFU) two weeks post-boost. After virus challenge, mice were daily examined for survival and clinical signs, and blood samples were collected at 3, 5, 7, 10 and 14 d.p.i. for the analysis of RNAemia by RT-qPCR.

### Viraemia and RNAemia analysis by plaque assay and RT-qPCR

Blood samples were collected from the submandibular plexus of mice with EDTA as anti-coagulant. For the analysis of viraemia by plaque assay, 50 µL of blood were diluted in PBS and centrifuged at 3000 rpm for 10 minutes. Thereafter, supernatant was removed, and pellet was lysed in 450 µL of sterile water for 2 minutes. Cell lysis was stopped by adding 50 µL of PBS10X. Then, samples were inoculated into 12-well plates containing semi-confluent monolayers of Vero cells. Following incubation for 1 h, an agar overlay (DMEM-10%-FBS-0.4%-Noble Agar, Becton Dickinson, MD, USA) was added and plates were incubated for 5 days at 37°C in 5% CO_2_. Plaques were fixed with 10% formaldehyde and visualized with 2% crystal violet-PBS.

For the analysis of RNAemia by RT-qPCR, RNA was extracted from 50 µL of blood using TRIzol Reagent (Sigma Aldrich, St. Louis, MO, USA) following the protocol established by the manufacturer. Viraemia was analyzed in duplicate by real-time RT-qPCR specific for EHDV segment 9 (encoding for VP6 and NS4). The real-time RT-qPCR specific for EHDV segment 9 was performed using primers and probe described by Maan *et al.* 93. Only Ct values lower than 38 were considered indicative of viremia (positive). “No Ct” values were considered as a Ct of 45, the last cycle of the RT-qPCR.

For the coinfection experiment, the BTV RNA in blood was analyzed in duplicate by real-time RT-qPCR specific for BTV segment 5 (encoding for NS1). The real-time RT-qPCR specific for BTV segment 5 was performed using primers and probe described by Toussaint *et al.* 94. Only Ct values lower than 38 were considered indicative of viremia (positive), according to the cut-off established by Toussaint *et al.* 94. “No Ct” values were considered as a Ct of 45, the last cycle of the RT-qPCR.

### Viral burden analysis in target organs by RT-qPCR

Organs analyzed (spleen, lung, liver, thymus, kidney, heart, testicles and epididymis) were collected, weighted, and homogenized in TRIzol reagent (Sigma Aldrich, St. Louis, MO, USA) with a BeadBug 6 homogenizer (Benchmark Scientific, TEquipment Inc. Sayreville, NJ, USA) in 2 mL centrifuge tubes containing 1.5 mm Zirconium beads. Total RNA was extracted from homogenized tissues following the protocol established by the manufacturer. RNA was analyzed in duplicate by real-time RT-qPCR specific for EHDV segment 9 (encoding for VP6 and NS4). The real-time RT-qPCR specific for EHDV segment 9 was performed using primers and probe described by Maan *et al.* 93. Data are expressed as PFU equivalents/g of tissue by comparison with previously titrated samples as previously described 67.

### Histopathology

Samples from different tissues and organs were taken and fixed in 10% buffered formalin (pH 7.2) for histopathological studies. After fixation, samples were dehydrated through a graded series of alcohol to xylol and embedded in paraffin wax. Sections of 4-im-thick were cut and stained with hematoxylin and eosin (H & E) for histopathological analyses.

### Blood measurements

A multiparameter autohematology analyzer (BC-5300 Vet; Mindray, China) was used to determine the total and differential cell counts in mice blood for each group collected into EDTA tubes.

### Analysis of cytokine gene expression and transcriptional regulation

To monitor proinflammatory cytokines (IL 1-β, IL-6, IL-12p40, IFN-γ, TNF and CXCL2), transcript levels in organs of IFNAR(-/-) mice infected with a lethal dose of EHDV-8 were quantified by RT-qPCR. RNA was reverse transcribed using High Retrotrasncriptase Starter Kit with Oligo dT (Biotools) to synthesize the first strand cDNA following the manu-facturer's instructions.

The relative quantification of pro-inflammatory cytokines was performed by the ∆Ct (Ct of gene of interest - Ct housekeeping gene) method using β-actin as a house-keeping gene and PrimeTime Std qPCR Assays (Integrated DNA Technologies): Mm.PT.39a.22214843.g for β-actin, Mm.PT.58.10005566 for IL-6, Mm.PT.58.12575861 for TNF-α, Mm.PT.58.41616450 for IL-1β, Mm.PT.58.10005566 for IFN-γ, and Mm.PT.58.10456839 for CXCL2. Amplification conditions were as follows: 50°C for 2min, 95°C for 10 min at, and 45 cycles of 15 sec at 95°C, and 60 sec at 60°C. Fluorescence data was acquired at the end step. RT-qPCR was performed on an Illumina ® ECOTM thermal cycler (ECOTM Real-Time PCR System, Illumina®).Results were expressed as fold change over the control (ΔCt EHDV infected/ΔCt mock). Samples with negative results in PCR that fell below the level of detection of the assay were assigned Ct 45. The mean ΔΔ CT values of duplicate samples of test and control mice were used for analysis. Results were presented as means ± standard error (SD) of data from duplicate replicates.

### Determination of circulating levels of cytokines

Sera from immunized and non-immunized mice were extracted the day before the challenge and at 3 and 5 d.p.i. Circulating cytokine levels were analyzed using a multiplex fluorescent bead immunoassay for quantitative detection of mouse cytokines (Millipore's MILLIPLEX Mouse Cytokine kit, Burlington, MA, USA). Samples were analyzed with a MAGPIX system (Luminex Corporation, Austin, TX, USA). Values of pre-challenge samples were subtracted from values of post-challenge samples.

### Plaque reduction neutralization test

Two-fold dilutions (from 1:5) of heat inactivated mice sera (56°C for 30 min) were incubated with 100 PFU of EHDV-6 or EHDV-8 for 1 h at 37°C. Then, samples were inoculated into 12-well plates containing semi-confluent monolayers of Vero cells. Following incubation for 1 h, an agar overlay (DMEM-10%-FBS-0.4%-Noble Agar, Becton Dickinson, MD, USA) was added and plates were incubated for 5 days at 37°C in 5% CO_2_. Plaques were fixed with 10% formaldehyde and visualized with 2% crystal violet-PBS. A 50% plaque reduction neutralization test (PRNT_50_) titer was calculated as the reciprocal (log_10_) of the highest dilution of serum that neutralized 50% of the control virus input. The cut-off is 0.69, log_10_ of the reciprocal of the first dilution 1:5.

### Statistical analysis

Data were analyzed using GraphPad Prism version 8.0.1 (GraphPad Software, San Diego, CA, USA). Survival curves for each immunized group were compared to those of non-immunized mice in search of statistical differences using Log-rank test. Comparisons of mean responses between groups in the viremia and RNAemia analysis were conducted by multiple t test analysis using the Sidak-Bonferroni method. Differences between groups regarding RNA levels in harvested organs were determined by Kruskal-Wallis test. It must be noted that “No Ct” values were converted to a Ct of 45 (last cycle of the specific RT-qPCR). Differences between groups regarding hematologic parameters, cytokine gene expression and circulating cytokine levels were analyzed using Mann-Whitney non-parametric test. A p-value lower than 0.05 was considered significant in all cases.
